# Update to: Assessing the efficacy of male *Wolbachia*-infected mosquito deployments to reduce dengue incidence in Singapore

**DOI:** 10.1186/s13063-024-08148-z

**Published:** 2024-06-20

**Authors:** Jue Tao Lim, Diyar Mailepessov, Chee-Seng Chong, Chia-Chen Chang, Borame Dickens, Yee Ling Lai, Lu Deng, Caleb Lee, Li Yun Tan, Grace Chain, Soon Hoe Ho, Muhammad Faizal Zulkifli, Jonathan Liew, Kathryn Vasquez, Vernon Lee, Judith Chui Ching Wong, Shuzhen Sim, Cheong Huat Tan, Lee Ching Ng

**Affiliations:** 1 Lee Kong Chian School of Medicine, Nanyang Technological University, Singapore, Singapore; 2 Environmental Health Institute, National Environment Agency, Singapore, Singapore; 3 Saw Swee Hock School of Public Health, National University of Singapore and National University Health System, Singapore, Singapore; 4 Ministry of Health, Singapore, Singapore; 5 School of Biological Sciences, Nanyang Technological University, Singapore, Singapore

**Keywords:** Dengue, *Wolbachia*, Cluster-randomised controlled trial

## Abstract

**Background:**

This trial is a parallel, two-arm, non-blinded cluster randomised controlled trial that is under way in Singapore, with the aim of measuring the efficacy of male *Wolbachia*-infected *Aedes aegypti* deployments in reducing dengue incidence in an endemic setting with all four dengue serotypes in circulation. The trial commenced in July 2022 and is expected to conclude in September 2024. The original study protocol was published in December 2022. Here, we describe amendments that have been made to the study protocol since commencement of the trial.

**Methods:**

The key protocol amendments are (1) addition of an explicit definition of *Wolbachia* exposure for residents residing in intervention sites based on the duration of *Wolbachia* exposure at point of testing, (2) incorporation of a high-dimensional set of anthropogenic and environmental characteristics in the analysis plan to adjust for baseline risk factors of dengue transmission, and (3) addition of alternative statistical analyses for endpoints to control for post hoc imbalance in cluster-based environmental and anthropogenic characteristics.

**Discussion:**

The findings from this study will provide the first experimental evidence for the efficacy of releasing male-*Wolbachia* infected mosquitoes to reduce dengue incidence in a cluster-randomised controlled trial. The trial will conclude in 2024 and results will be reported shortly thereafter.

**Trial registration:**

ClinicalTrials.gov, identifier: NCT05505682. Registered on 16 August 2022. Retrospectively registered. Last updated 11 November 2023.

## Update

This update relates to the study protocol for a cluster randomised controlled trial (cRCT) to evaluate the efficacy of male *Wolbachia*-infected mosquito deployments to reduce dengue incidence in Singapore. This update should be read in conjunction with the original protocol publication [1].

## Definition of *Wolbachia* exposure

The original protocol did not explicitly define the inclusion criteria of residents based on the duration of *Wolbachia* exposure at the point of dengue testing. Preliminary field trial data from sites prior to the commencement of the cRCT [2] demonstrated that at least 3–12 months was required for the intervention to demonstrate noticeable suppression in *Aedes aegypti* abundance.

For the first primary endpoint, we aimed to examine the intervention efficacy of *Wolbachia* interventions to reduce the risk of contracting dengue using a test-negative design. To understand any potential relationship between the duration of intervention and the risk of contracting dengue at the point of testing, we will consider participants to be exposed/unexposed to *Wolbachia* in the study based on whether they were subject to 0, 3 or 6 or more months of intervention or residing in a control site respectively at the time of testing in the post-intervention period. Higher exposure durations will be considered if sample size is sufficient for analyses. Exposed and unexposed participants from the same exposure duration will be compared.

Similarly, for the second primary endpoint of examining the efficacy of *Wolbachia* to reduce town-level dengue case counts normalised by cluster population size, we will re-aggregate intervention efficacies based on 0, 3 or 6 or more months of intervention for intervention sites to compare the reduction in dengue incidence versus controls in the post-intervention period. A further re-aggregation based on calendar time would also be explored following previous work in the study setting showing that intervention efficacies may be confounded by whether it was a national inter-epidemic or epidemic year [3, 4] and to remove temporal confounding effects from analyses.

For the secondary endpoint of examining the efficacy of *Wolbachia* to reduce town-level *Aedes aegypti* abundance, we will re-aggregate intervention efficacies based on 0, 3 or 6 or more months of intervention for intervention sites to compare the reduction in *Aedes* abundance versus controls in the post-intervention period.

## Addition of supplemental environmental and anthropogenic data to control for baseline dengue risk in intervention and control arms

We additionally extracted a set of spatially and temporally explicit variables to represent environmental heterogeneity across the study sites which was previously not available to the study team. The data would be used to explore and adjust for imbalances in variables which may potentially confound historical dengue risk, using propensity scoring models as described in the revised statistical analysis below. Several of these factors were previously found to be associated to *Aegypti* abundance and dengue transmission from previous baseline studies in the same study setting [5–7].

1. A 10-m vegetation map [8] with areas classified across multiple vegetation types including grass, forest and managed vegetation based on Sentinel-2 satellite data was utilised to signify the availability of natural breeding sites and nectar availability for mosquito males. The percentage cover of each vegetation type was calculated within each sector as mosquitoes often show preferential areas to breed and rest. Similarly, the averaged Landsat Normalized Difference Vegetation Index per sector was also utilised for this purpose [9].
2. To represent both host density and urban breeding habitat availability, data on the locations of Housing and Development Board (HDB) public housing estates where over 80% of Singapore’s resident population reside was obtained from Onemap [10]. Utilising the HDB location and HDB resale data, the average age of HDB buildings was collected as older age is a well-established risk factor for higher Gravitrap indices [5, 7]. This is due to building deterioration providing additional breeding habitats in cracks and design features such as laundry poles which are no longer built due to the pooling of water within the supports. Average HDB house price from 2015 to 2022, a proxy for household income and socioeconomic status, was calculated based on an XGBoost model previously employed [11]. Building height, which has also been correlated to Gravitrap indices, was calculated according to the number of floors and average height of each level of 3 m. The number of condominiums and landed properties was additionally collected within each sector representing additional hosts being available. The percentage cover of built area was calculated as a sum of all residential, commercial and industrial buildings, representing the level of urbanicity, which has been associated with *Ae. aegypti* presence [12]. The major open drainage network for Singapore was obtained from the Public Utilities Board as a key breeding site for mosquitoes around HDBs. The average distance of each HDB block within a sector to a drain was measured as well as the length of the network within the sector [6].
3. For meteorological data, well-established variables which are known to affect mosquito survival or fecundity were collected. These included daily mean, maximum, and minimum temperature, total rainfall, maximum rainfall falling within a 30-min, 60-min and 120-min window, and wind speed, which were obtained from a total of 21 weather stations installed by the National Environment Agency. We created daily complete raster maps through inverse distance weighting interpolation, which was carried out using cross validation of leave-one-out for the fitting of the inverse distancing power to minimise the error in observation on the raster surface of the test point. Hourly dewpoint and ambient ground air temperature were taken from ERA5, published by ECMWF [13], to estimate relative humidity over the time period using standard formula. These values were aggregated at a weekly level to correspond with the dengue case data.

## Update on pre-post trial attitudes, acceptance, and knowledge survey on *Wolbachia-Aedes* interventions and other vector control practices

Household surveys conducted at baseline and after 3 months of engagement has yielded sufficient data to meet the objective of assessing the efficacy of various engagement strategies employed; hence, the survey at 1 year post-commencement will not be conducted. Survey findings showed increased awareness in all arms and knowledge gaps were similar to previous surveys (2016 and 2019). In addition, the level of trust and acceptance of the project remain high and consistent with previous surveys. A limitation to the study was that site selection for the engagement arms was not randomised in view of ground considerations. The engagement strategies used in the intervention arms were also difficult to maintain and evaluate beyond 3 months as it was confounded by the varying intensity of ground up efforts by community partners.

## Revised supplemental statistical methods for first primary endpoint

To account for any residual imbalance in anthropogenic and environmental characteristics, we will explore the utilisation of either inverse-probability weighting, the doubly robust logistic regression or overlap weighting to account for post hoc imbalance in aforementioned spatio-temporal characteristics.

Secondary cluster-level analysis would also now consider the computation of weighted odds ratios to understand the reduction in odds of being dengue-test positive by residing in a *Wolbachia* intervention site for a specified amount of time, with inference either being conducted using the permutation inference or cluster bootstrap approach [14]. We will similarly explore utilising either inverse-probability weighting or overlap weighting to account for post hoc imbalance in aforementioned spatio-temporal characteristics.

Standardised mean differences will be used to compare balance in unweighted and weighted characteristics in the pre-intervention period in intervention and control arms.

## Revised supplemental statistical methods for second primary endpoint and secondary endpoint

In the proposed difference-in-difference (DiD) statistical methodology, incorporation of anthropogenic and environmental characteristics will also be explored. Weighted DiD would be explored as an alternative analytical approach if town-level anthropogenic and environmental characteristics were not well-balanced prior to intervention. Weights will potentially be estimated either using inverse-probability weighting or overlap weighting at the town level to balance anthropogenic and environmental characteristics. The weights will then be included in the regression models prior to estimation of intervention efficacies.

Standardised mean differences will also be used to compare balance in unweighted and weighted characteristics in the pre-intervention period at the town-level between intervention and control arms.
